# Synergistic Manipulation of Zero-Dimension and One-Dimension Hybrid Nanofillers in Multi-Layer Two-Dimension Thin Films to Construct Light Weight Electromagnetic Interference Material

**DOI:** 10.3390/polym13193278

**Published:** 2021-09-26

**Authors:** Bihui Jin, Feiran Meng, Haoyu Ma, Bowen Zhang, Pengjian Gong, Chul B. Park, Guangxian Li

**Affiliations:** 1 State Key Laboratory of Polymer Materials Engineering, College of Polymer Science and Engineering, Sichuan University, 24 Nanyiduan of Yihuan Road, Chengdu 610065, China; bihuijin@yeah.net (B.J.); feiranmeng@yeah.net (F.M.); haoyuma10@163.com (H.M.); bowen1019@outlook.com (B.Z.); 2 Institute of Advanced Polymer Materials Technology, JITRI, Nanjing 210000, China; 3 Microcellular Plastics Manufacturing Laboratory, Department of Mechanical and Industrial Engineering, University of Toronto, 5 King’s College Road, Toronto, ON M5S 3G8, Canada; park@mie.utoronto.ca

**Keywords:** hybrid conductive nanofillers, multi-dimension structure, EMI shielding, low EM reflection, nanocomposite foam

## Abstract

Nanocomposite foam with a large expansion ratio and thin cell walls is promising for electromagnetic interference (EMI) shielding materials, due to the low electromagnetic (EM) reflection and high EM absorption. To overcome the dimensional limitation from two-dimension (2D) thin walls on the construction of conductive network, a strategy combining hybrid conductive nanofillers in semi-crystalline matrix together with supercritical CO_2_ (scCO_2_) foaming was applied: (1) one-dimension (1D) CNTs with moderate aspect ratio was used to minimize the dimensional confinement from 2D thin walls while constructing the main EM absorbing network; (2) zero-dimension (0D) carbon black (CB) with no dimensional confinement was used to connect the separated CNTs in thin walls and to expand the EM absorbing network; (3) scCO_2_ foaming was applied to obtain a cellular structure with multi-layer thin walls and a large amount of air cells to reduce the reflected EM; (4) semi-crystalline polymer was selected so that the rheological behavior could be adjusted by optimizing crystallization and filler content to regulate the cellular structure. Consequently, an advanced material featured as lightweight, high EM absorption and low EM reflection was obtained at 0.48 vol.% hybrid nanofillers and a density of 0.067 g/cm^3^, whose specific EMI shielding performance was 183 dB cm^3^/g.

## 1. Introduction

Nowadays, with the increasing demand of high-performance communication equipment in industry, medical treatment, military and many other aspects, the transmission of electromagnetic (EM) waves especially at high frequency become important [1]. However, electromagnetic interference (EMI) causes the loss of information or even misinformation, the higher the signal frequency, the more serious the interference [2,3]. Therefore, EMI shielding materials are an important issue to guarantee the high performance and signal integrity in communications equipment [4]. The way that EMI shielding material works is to reflect and absorb EM repeatedly to block the transmission of a redundant signal [5]. Along with increasing bandwidth in mobile communication equipment, a high frequency signal requires less reflective. This means the advanced EMI shielding materials absorb the incident redundant signal in very high efficiency but reflect the incident redundant signal in an extremely low efficiency. Due to this the redundant signal could be fully absorbed by the EMI shielding material and be blocked from both transmission (protecting other signal emitting elements from signal interference) and reflection (maintaining the signal stabilization of the signal emitting element) [6,7,8]. The high-end mobile communication equipment of signal integrity, accuracy and reliability is calling for advanced lightweight EMI shielding materials with strong EM absorption and low EM reflection [9,10].

Conductive nanocomposites have many advantages, such as lightweight, easy processing, chemical corrosion resistance, structure-property controllable, etc. These features enable them to widely replace traditional metal or ceramic materials in EMI shielding [11,12,13,14], fuel cell bipolar plate [15,16], dielectric for energy storage [17,18], electrostatic protection [19] and many other application fields. High electric one-dimension (1D) carbon nanotubes (CNTs) are frequently added to polymer matrix as electrical conductive particles as CNTs have a high aspect ratio and then form an electrical conductive network structure in a low threshold content [20]. High electrical conductivity makes the nanocomposites absorb the incident EM signal and then convert this into current (electricity) before finally being consumed as Joule heat (heat) [21]. However, electrical conductive nanocomposites also reflect incident EM waves.

Cellular structure is also widely introduced for nanocomposites, to regulate the dispersive status of nanofillers and to control the performance correspondingly [22,23,24]. In conductive nanocomposite foam, the dielectric constant is supposed to be reduced to near air when there is a large amount of air, and hence the reflection of incident EM signal decreases because there is less impedance mismatch between the air and the material [8]. Furthermore, when the large amount of air is separated into independent cells by thin films whose thickness is much smaller than the wavelength of the incident EM signal, an interference phenomenon occurs at the surface of thin films to block EM reflection [25]. The transmitted EM signal is then absorbed by the three-dimension (3D) electrical conductive network formed in multi-layer two-dimension (2D) thin films constructed from conductive nanofillers (e.g., 1D CNTs).

Due to the complicated structure of conductive nanocomposite foam, that is multi-scale, multi-dimension and multi-component (3D micro-scale gas phase supported by continuously connected 2D solid matrix embedded with 1D nano-scale conductive fillers). It is extremely difficult to clarify the relationship between EMI shielding performance and material structure [26]. Although the electrical conductive network plays a key role in EMI shielding, but a distinct influence from cellular structure in nanocomposite on electrical conductivity is still unclear. Xu et al. [27] prepared polyurethane (PU)/CNT foam, they found that the conductivity of PU/2 wt% CNT decreased from 2 × 10^−2^ S/m to 4 × 10^−5^ S/m when the expansion ratio increased from 2.6 to 26.3 times. Tran et al. [28] prepared polypropylene (PP)/CNT foam with high conductivity and EMI shielding performance. The conductivity of obtained foams has a similar trend to that of PU/CNT foam prepared by Xu et al., the conductivity decreased with increasing expansion ratio. However, Ameli et al. [29] prepared PP/CNT foam using the CNTs with a similar size to Tran (and the same foaming method), the conductivity of PP/CNT foam increased first and then decreased with increasing expansion ratio. Ameli et al. [30] also prepared PP/CNT foam by injection foam molding. They found that the electrical conductivity increased when cellular structure was introduced to the nanocomposite. Based on the research, it is noted that the dispersive status of CNTs in nanocomposite is significantly changed when cellular structure is introduced into nanocomposite. Therefore, foam’s conductivity is sole determined by the final status of CNTs in the foam.

In the foaming process of the polymer matrix containing CNTs, the cell wall stretching leads to CNTs’ orientation in cell walls along the stretching direction [31,32]. Therefore, the electrical conductive network based on connected CNTs became very different from that in the original matrix. When focused on CNTs in the nanocomposite foam, 1D CNTs are selectively distributed in the 2D cell walls and then continuously arranged along the cell walls in 3D space to form a cellular structure. This means the continuously connected CNTs’ conductive network also has a segregated structure centered by air cells, which makes the CNTs confined in 2D thin films between two adjacent cells. Therefore, it is necessary to make full usage of the synergistic effect of 1D CNTs, 2D thin films and 3D cells to effectively improve the EMI shielding performance.

Due to the dimensional confinement of 2D thin film, the originally random oriented CNTs in nanocomposite matrix will re-orient along the stretching direction. This means the dimension perpendicular to thin film thickness direction is confined. Such a dimensional confinement to CNTs then weakens the construction of the conductive network and correspondingly deteriorates the EMI shielding performance of CNT nanocomposite foams. Mixed conductive fillers are often applied to nanocomposite foams. Duan [33] et al. added CNTs or acetylene black (ACET) into epoxy/nickel coated carbon fiber (EP/NCCF) foam. They found that the mixed conductive fillers had better EM blocking effect than only adding carbon fiber. Wu et al. [34] added both CNTs and carbon black (CB) and found that the mixed conductive filler had almost no synergistic effect in the nanocomposite, until air cells were introduced into the nanocomposite and then a synergistic effect was observed. Ju et al. [35] also added CNTs and CB to nanocomposite foam for better performance. In the case of EMI shielding materials with lightweight, high EM absorption and low EM reflection characteristics, a large amount of air has to be added to the nanocomposite together with the formation of multi-layer thin cell walls. This means the mixed conductive fillers would go through a strong dimensional confinement. Although mixed conductive fillers show a certain synergistic effect in nanocomposite foam with a low expansion ratio (limited dimensional confinement), how the system works in largely expanded nanocomposite foam to obtain the advanced EMI shielding materials, especially with a low EM reflection, is still unclear. The key point is to identify the dispersive status of mixed conductive fillers in foams and the corresponding relationship between conductive network and cellular structure.

In this work, hybrid carbonaceous nanofillers of 1D CNT and 0D CB were added to a semi-crystalline random copolymer. Nanocomposite foams were then prepared by scCO_2_ foaming. The conductive nanocomposite foams with large expansion were obtained by adjusting the foaming process. Combining the experimental tests and numerical simulation, it is revealed that the dimensional limitation to 1D CNTs from 2D thin cell walls is detrimental to the formation of a conductive network. By adding 0D CB particles, the adverse effect from dimensional limitation (1D CNTs in 2D thin film) was overcome and a lightweight conductive nanocomposite foam with a low percolation threshold, high EM absorption and low EM reflection was successfully achieved. When the volume content of hybrid conductive nanofillers is 0.48 vol.%, the obtained EMI shielding material had a specific EMI shielding performance as high as 183 dB cm^3^/g with 97% absorption and only 3% reflection and a density as low as 0.067 g/cm^3^.

## 2. Materials and Methods

### 2.1. Materials

Random copolymer polypropylene (PPR, density is 0.9 g/cm^3^, Sinopec Inc., Zhenhai, China), multi-walled carbon nanotubes (MWCNTs, NC7000, purity ≥ 90%, diameter is 9.5 nm, length is 1.5 μm, JOUWU Inc., Shanghai, China), conductive carbon black (resistance ≤ 1.5 Ω, Tianjin Zhengningxin Material Technology Co., Ltd., Tianjin, China), carbon dioxide (purity ≥ 99%, Linde Gas, The Linde Group Inc., Shanghai, China) was used as the physical blowing agent.

### 2.2. Preparation of Nanocomposites

PP was dried at 70 °C for 6 h in a vacuum oven (DZF-6050 AB) before processing to reduce moisture. PP pellets were then mixed with CNTs and/or CB in a torque rheometer (XSS-300) at a condition of 190 °C and 60 r/min for 5 min to obtain the nanocomposites. Nanocomposites were then pressed into round plates with a diameter of 25 mm and a thickness of 1.1 mm for rotary rheological testing, or into long strip plates with a size of 30 mm × 10 mm × 1.1 mm for foaming and electrical properties testing.

### 2.3. Preparation of Nanocomposite Foam

Nanocomposites were placed in an autoclave for 2 h at the condition of 145 °C and 15 MPa supercritical carbon dioxide (scCO_2_). The pressure was quickly released to initiate cell nucleation followed by cell growth. Nanocomposite foam with a variety of cellular morphology could be obtained by foaming at different temperatures and pressures. Long strip foam plates with the size of 20 mm × 5 mm × 3 mm was prepared for electrical properties testing. The foaming process is shown in Supporting Information (SI).

### 2.4. Characterization

#### 2.4.1. Scanning Electron Microscopy

Scanning Electron Microscope (SEM, Phenom Pro) was used to study the cellular structure of nanocomposite foams. Before SEM characterization, the sample surface was coated with gold. SEM micrographs were then collected at an acceleration voltage of 25 kV using SEM. Using *ImageJ* software, the average size of the cell (average of 300 cells) can be summarized. The cellular structure data of PP/CNT, PP/CNTCB and PP/CB conductive nanocomposite foams were then obtained. Cell wall thickness can be obtained by the conversion formula from cell size and expansion ratio [36].

#### 2.4.2. Rheology

A rotary rheometer (DHR-3) was used to study the rotational rheological properties of nanocomposites. Testing was conducted in a range of 0.1–100 rad/s at 250 °C and a strain amplitude of 0.15% to analyze the rheological network structure constructed by carbonaceous fillers. Circular plate samples with a diameter of 25 mm and a thickness of 1.1 mm were used.

#### 2.4.3. Thermal Property

Differential scanning calorimetry (DSC, 250) was used to test the thermal properties of both bulk and foam samples to obtain the melting temperature, crystallization temperature and crystallinity. Testing conditions were set as follows: sample was first heated from 40 °C to 200 °C at a heating rate of 10 °C/min, then stayed in an isothermal state for 5 min to eliminate the thermal history, after that cooled to 40 °C at a cooling rate of 10 °C/min. The sample was then heated for a second time from 40 °C to 200 °C at a heating rate of 10 °C/min.

#### 2.4.4. Electrical Conductivity

A high resistance meter (SM7110) was used to study the conductivity of nanocomposites and foams. All samples were tested at least 3 times to obtain the average conductivity. The conversion formula based on additive volume content to calculate the resistance is included in SI.

#### 2.4.5. Electromagnetic Interference Shielding

A PNA-X network analyzer (Keysight N5247A) was used to characterize the EMI property of nanocomposites and foams in the X-band frequency range (8.2–12.4 GHz). Scattering parameters (*S*_11_ and *S*_21_) were used to calculate the total *SE* (*SE_T_*), reflection *SE* (*SE_R_*) and absorption *SE* (*SE_A_*). The detailed calculation method can be found in SI.

## 3. Results and Discussion

### 3.1. Rheological Property

The carbon fillers in the polymer matrix constructed a rheological network, which directly suppresses the movement of molecular chains, enhances the viscoelasticity of the polymer matrix [37], limits the cell growth and correspondingly affect the electrical conductivity of obtained foams.

**Figure 1:**
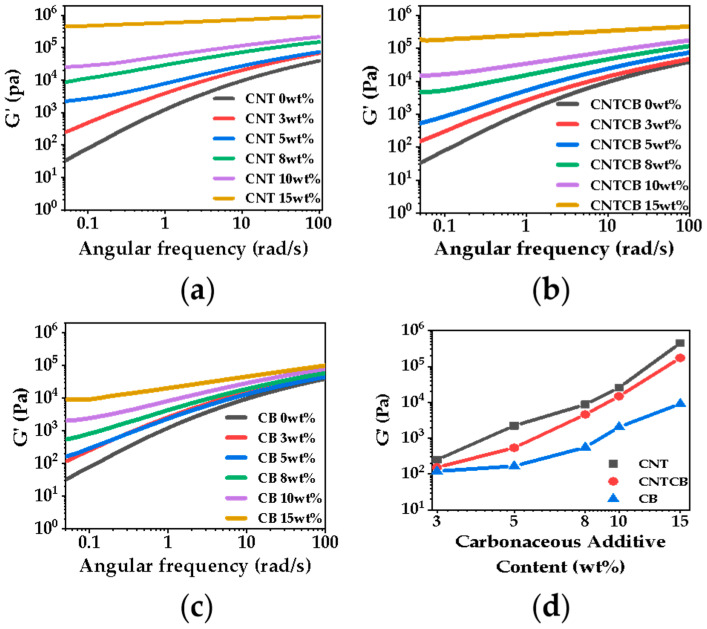
Relationship between frequency and storage modulus of nanocomposites with various carbonaceous filler contents: (**a**) PP/CNT; (**b**) PP/CNTCB; (**c**) PP/CB; (**d**) relationship between carbon filler content and storage modulus of nanocomposites at 0.05 rad/s.

Figure 1 shows the relationship between the storage modulus and frequency of nanocomposites. As CNT has a large aspect ratio, it has a greater advantage than CB in constructing the rheological network. With increasing filler content, a plateau appeared in the CNT system with 5 wt% filler content; a plateau appeared in the CB system with 10 wt% filler content; while a plateau appeared in the CNT/CB system with 8 wt% filler content. Compared with the CNT system, CB has less influence on the rheological network, so it has less influence on cell growth in the subsequent foaming process. This is conducive to the preparation of conductive nanocomposite foam with a large expansion ratio.

### 3.2. Thermal Property

**Figure 2:**
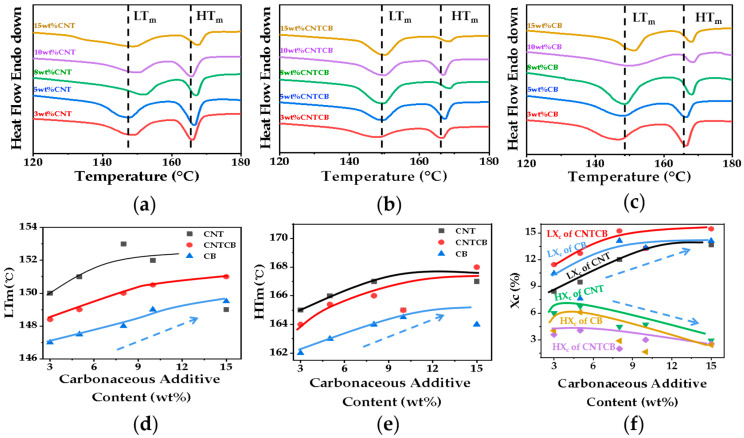
DSC melting curves of nanocomposite foams: (**a**) PP/CNT; (**b**) PP/CNTCB; (**c**) PP/CB; (**d**) first melting temperature; (**e**) second melting temperature; (**f**) crystallinity. *L* means low melting peak and *H* means high melting peak.

The foaming behavior of PP can be regulated by the foaming temperature because the residual crystals in the partial melting status can be used as a physical cross-linking point to improve the matrix strength and to stabilize the cell growth. Introducing the ethylene fragments into the PP macromolecular chains can reduce the regularity of the PP molecular chain, thus widening the melting range of the PP matrix and regulating its foaming behavior in a wider foaming temperature window. Therefore, although adding the carbon fillers will form a rheological network to hinder the cell growth, the effect of the CB on the matrix viscoelasticity is smaller than that of the CNTs. Furthermore, the influence from adding carbonaceous fillers on the matrix rheological behavior can be balanced by melting more crystals, so there is less of a physical cross-linking point. It is noted from Figure 2 that the melting peaks of conductive nanocomposite foam shift to higher temperatures with increasing filler content because the existence of the rheological network improves the rigidity of PP macromolecular chains and hence the crystals are melted at a higher temperature.

### 3.3. Cellular Structure of Nanocomposite Foams

**Figure 3:**
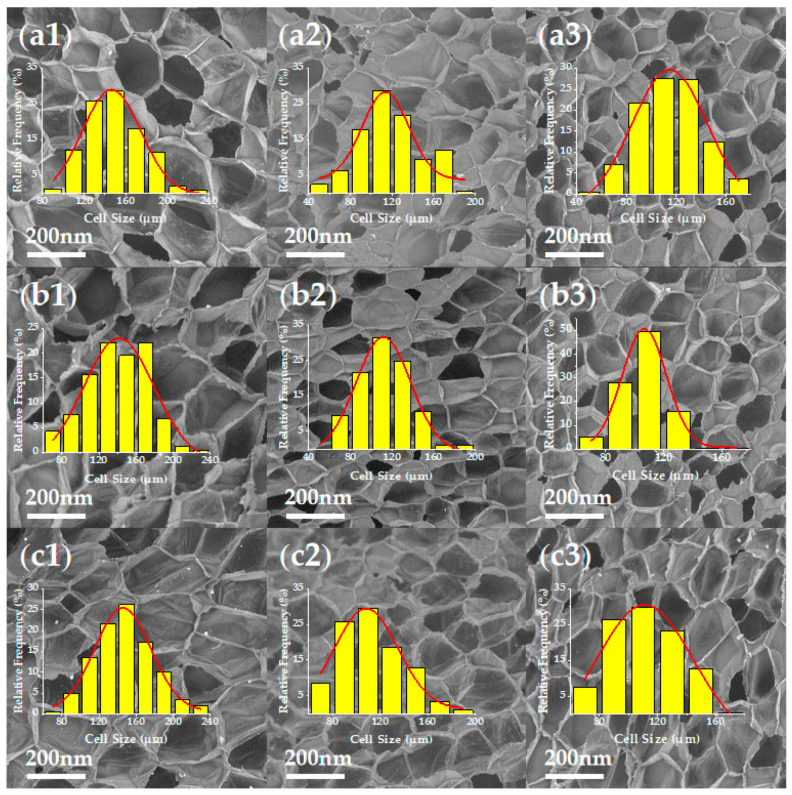
SEM micrographs of nanocomposite foams: (**a1**–**a3**) 5 wt%, 10 wt% and 15 wt% CB; (**b1**–**b3**) 5 wt%, 10 wt% and 15 wt% CNTCB; (**c1**–**c3**) 5 wt%, 10 wt% and 15 wt% CNT.

The cellular structure of nanocomposite foams is shown in Figure 3. (Other SEM micrographs of PP/CNTCB conductive nanocomposite foams at various foaming conditions can be found in SI).

**Figure 4:**
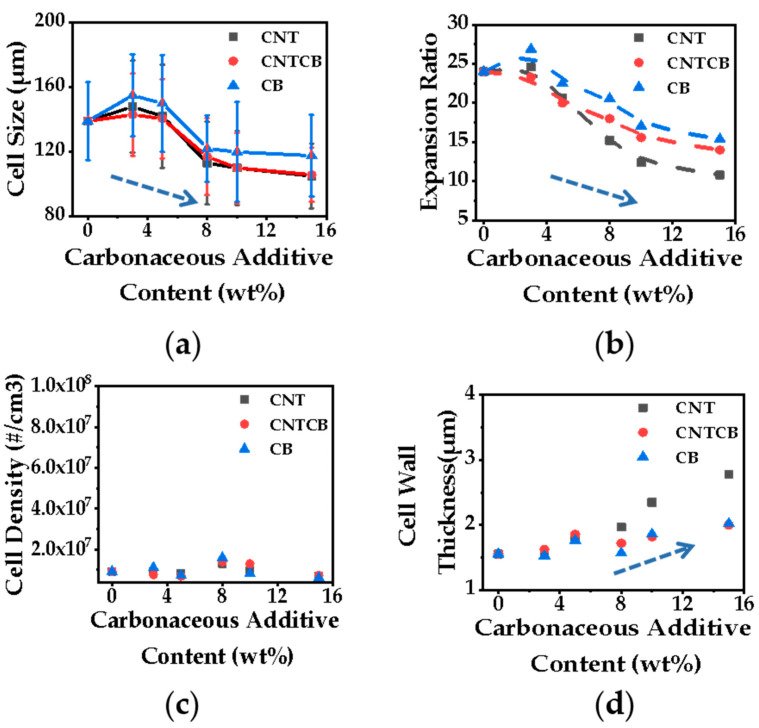
Cellular structure of PP/CNT, PP/CNTCB and PP/CB; (**a**) cell size; (**b**) expansion ratio; (**c**) cell density; (**d**) cell wall thickness.

Figure 4a–d shows the cellular parameters of nanocomposite foams (@145 °C &15 MPa)–PP/CNTCB’s cellular structure prepared at various foaming conditions can be found in SI. The nanofiller increases the heterogeneous interface, which is conducive to cell nucleation. However, the natural heterogeneous interface between the crystalline region and the amorphous region in semi-crystalline polymers weakens the cell nucleation ability of the heterogeneous interface from fillers. As can be seen from Figure 4c, cell density hardly changed with the filler content. In terms of cell growth, the cell size decreases with increasing filler content because the addition of filler increases the viscoelasticity of the system. It is extremely easy to construct the rheological network using CNT with a large aspect ratio so the degree of obstruction to cell growth is CNT > CNTCB > CB. Since the expansion ratio is affected by cell density and cell size, because the cell density is similar for all foams, the expansion ratio then shows a similar trend to cell size: the higher the filler content, the lower the expansion ratio; the higher the CNT content, the lower the expansion ratio, as shown in Figure 4a,b. The cell wall thickness is directly related to cell size and expansion ratio. When the cell density is constant, cells grow larger, cell walls are stretched to a higher degree (become thinner) and the foam is expanded, as shown in Figure 4d.

### 3.4. Effect of Hybrid Conductive Nanofillers and Cellular Structure on the Electrical Properties

Nanocomposite foam added by hybrid conductive nanofillers presents multi-scale (nano-scale fillers distributed in micro-scale cell walls to form the macro-scale conductive nanocomposite foam) and multi-dimension (0D CB and 1D CNTs distributed in 2D cell walls which connected with each other in 3D foam) and multi-component (air phase separated by a solid matrix embedded with conductive filler phase) features. Although the complicated structure enables a synergistic effect to construct the conductive network, it is extremely difficult to clarify how the synergetic effect works and then which effect plays the key role.

**Figure 5:**
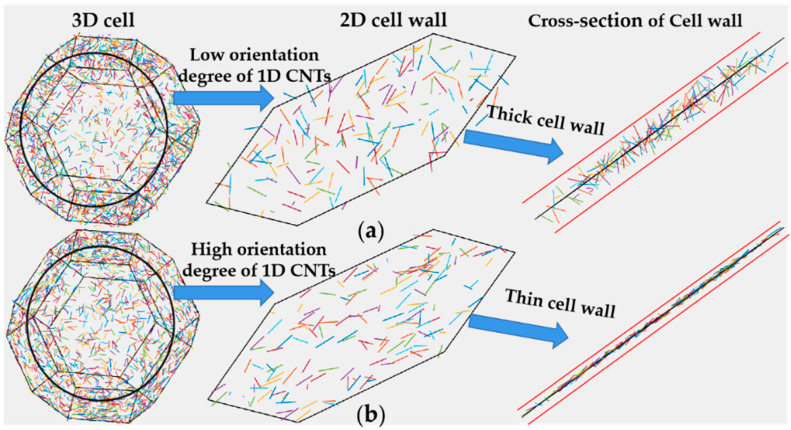
Confined distribution of 1D CNTs in 2D cell wall: (**a**) thick cell wall; (**b**) thin cell wall.

In nanocomposite foam containing conductive nanofillers, the dispersive status of conductive nanofillers is affected by the cellular structure because nanofillers only exit in cell walls. On the other hand, the cellular structure is affected by the conductive nanofillers because the viscoelasticity of nanocomposite is significantly altered by the conductive nanofillers, especially the 1D CNTs with a large aspect ratio that easily form the rheological network structure in a polymer matrix. Furthermore, in the foaming process, conductive nanofillers in cell walls rearrange along with cell wall stretching, resulting in a confined distribution in cell walls as shown in Figure 5. Therefore, the conductive network composed of conductive nanofillers such as CNTs is markedly affected by the cellular structure (e.g., cell size, cell density and cell wall thickness). Meanwhile, conductive nanofillers with different geometric structures are distributed in different confined states in cell walls so as to form a different conductive network in the cell wall.

**Figure 6:**
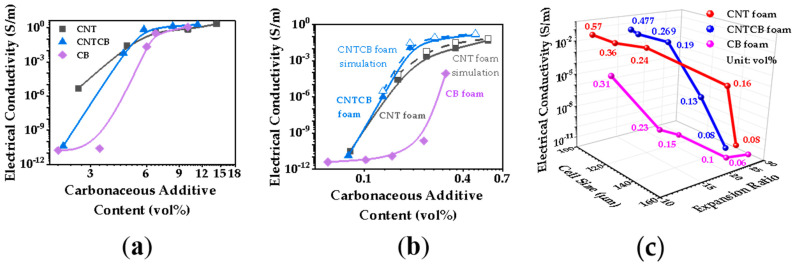
Electrical conductivity of nanocomposites (**a**) and foams (**b**) as a function of carbon filler content; (**c**) effect of cell size and expansion ratio on electrical conductivity.

Figure 6a,b compares the electrical conductivity of nanocomposites and their foaming materials at various filler contents. Compared with CB, CNT’s large aspect ratio makes it easier to construct conductive networks in nanocomposites. Therefore, the percolation threshold of CB composite was 4.5 vol.%, and then decreased to 3 vol.% after adding CNTs with CB at a ratio of 1:1. The percolation threshold was further reduced to below 2 vol.% when the conductive network was constructed entirely by CNTs. When cellular structure was introduced into the nanocomposite, the tensile orientation of the cell walls significantly weakened the CNTs’ ability of constructing the conductive network, because large aspect ratio CNTs were confined in thin cell walls. Since CNTs changed from 3D random distribution to restricted within film orienting distribution, the CNT conductive network was destroyed and the electrical conductivity decreased [31]. With the cellular structure introduced into the nanocomposites, filler contents were also markedly reduced. It is a positive factor to improve electrical conductivity. Although CB is much less capable of constructing conductive networks than CNTs both have 3D random distribution and confined orienting distribution, as shown in Figure 6a,b. Adding CB with CNTs enhanced conductivity in confined orienting distribution as shown in Figure 6b, indicates that CB and CNTs did have a synergistic effect in the restricted distribution state in cell walls, and effectively improved the conductivity of nanocomposite foam. Furthermore, the conductivity of nanocomposite foam is also affected by the cellular structure. Figure 6c shows the conductivity of materials with a variety of cellular structure. It is noted that the larger the expansion ratio, the lower the concentration of carbonaceous filler per unit volume, and the lower the electrical conductivity.

**Figure 7:**
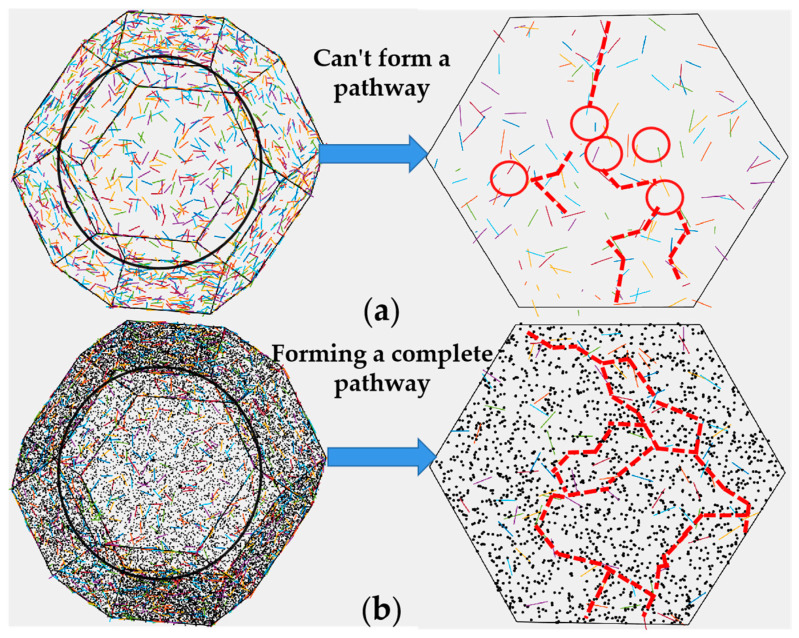
Schematic diagram of conductive path constructed by CNTs and/or CB at the same carbonaceous additive content (vol.%); (**a**) CNT; (**b**) CNTCB.

**Figure 8:**
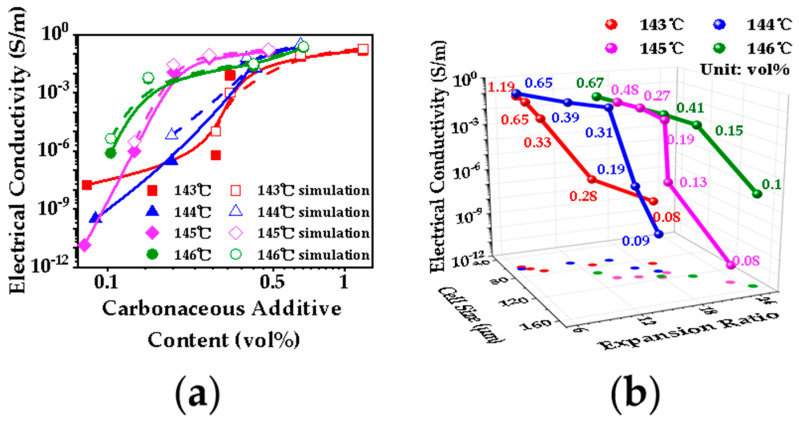
Electrical conductivity of PP/CNTCB foams (**a**) and the relationship between cell size and expansion ratio (**b**).

To further analyze the synergistic effect of CNTs and CB in the thin cell wall, we constructed a cell model with conductive nanofillers distributed in the cell wall. In the foaming process, conductive nanofillers are oriented along the stretching direction of cell walls, so they are in a confined status in cell walls. The cell wall thickness of nanocomposite foams was only 1–3 μm as shown in Figure 4d (much less than the length of CNTs). CNTs presented the confined distribution in cell walls, as shown in Figure 7a. Cell wall stretching makes CNTs in cell walls move far away from each other, resulting in the shortest distance among CNTs becoming larger than the electron tunneling distance. Therefore, the conductive network constructed in the nanocomposite is destroyed in the process of cell wall stretching. As CB is less affected by the confinement in cell walls, it is added to the cell cavity model. At equal proportions of CNT and CB, the CNTCB system is much easier to build a conductive network in thin film than the CNT system despite the fact the CNTCB system has a smaller CNT number than the CNT system. Based on the cell model and the dispersive status of conductive fillers, the conductivity of foam was calculated and the results are shown in Figure 6b and Figure 8a. The calculated results were very close to the experimental results, indicating that the assumption in the model that CNTs are confined in cell walls and CB cooperates with the oriented CNTs to construct the conductive network is correct.

To explore the influence of cell structure on volume conductivity for PP/CNTCB foam, we developed a novel lightweight EMI shielding material with low percolation threshold and high EM absorbability. Various cell structures were obtained by adjusting the foaming process, and the results are shown in Figure 8. At low foaming temperatures (143 °C and 144 °C), the expansion ratio of the nanocomposite is low and the cell size is small due to the existence of a large number of crystals. Due to the low tensile degree of the cell wall and the high content of conductive filler per unit volume of foam material, nanocomposite foam shows high conductivity. At high foaming temperatures, a small number of crystals exist, and cell growth becomes easier. Although the tensile degree of the cell wall is high, the presence of CB makes the CNTs oriented along the tensile direction of cell walls have a good connectivity with each other. This partially offsets the negative effect caused by the reduction of the content of conductive filler per unit volume of foam material. Therefore, the conductivity of nanocomposite foams decreases slowly.

### 3.5. Electromagnetic Shielding Property of Nanocomposite Foams

**Figure 9:**
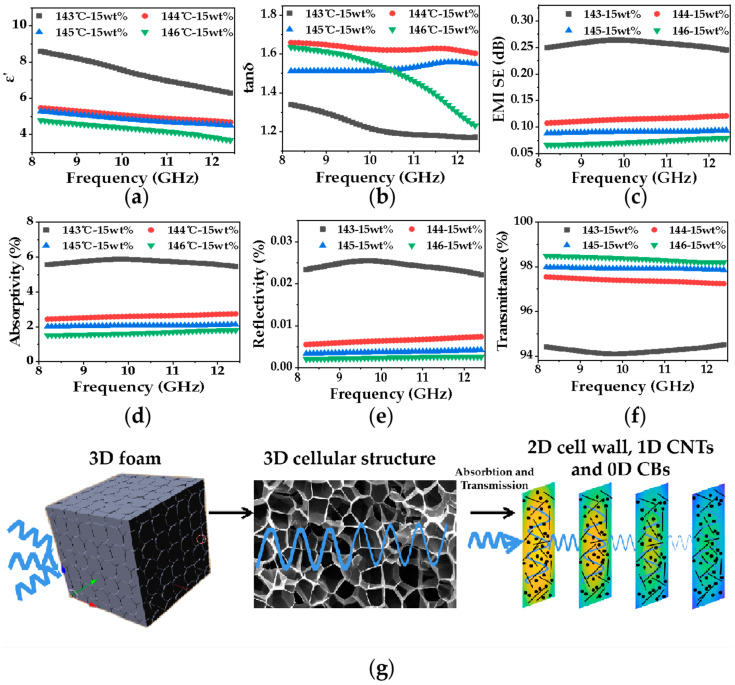
Dielectric property and EMI of single cell wall in PP/CNTCB conductive nanocomposite foams at different foaming temperatures; (**a**) complex permittivity; (**b**) dielectric loss; (**c**) EMI SE of single cell wall; (**d**) absorptivity of single cell wall; (**e**) reflectivity of single cell wall; (**f**) transmittance of single cell wall; (**g**) transmission process of EM wave through foam.

Introducing a large amount of air into nanocomposites can reduce the difference of wave impedance between nanocomposite foam and air, thus reducing the reflection of the EM wave. As the thickness of the continuous 2D cell walls inside the foaming material is much smaller than the incident EM wavelength, the reflection interference phenomenon generated makes the reflected EM wave decrease, hence the transmitted EM wave increases. This further weakens the EM wave’s reflection of nanocomposite foam. When a large number of conductive particles distributed in cell walls form a conductive network, electrons can form a macroscopic current along the conductive network driven by the EM field, and finally convert the incident EM wave into joule heat. The less EM wave reflected, the more EM wave entering nanocomposite foam, and the more EM wave absorbed. Nanocomposite foam has a large number of cell walls in the thickness direction of millimeter scale, so the EM wave transmitted is absorbed by a large number of layers until it is nearly completely shielded, as shown in Figure 9g.

When EM waves pass through the multi-layer thin walls in the nanocomposite foam, the 3D conductive network in foam constructed by hybrid CNTs and CB in thin cell walls can fully absorb incident EM waves. The dielectric parameters of the PP/CNTCB conductive nanocomposite with multi-layer continuous thin walls were then obtained by removing the air phase, as shown in Figure 9a,b. Figure 9c–f shows the calculated EM interference shielding, absorption, reflection and transmission performance of one single layer thin wall based on the obtained dielectric properties. For foam prepared at 143 °C, since the cell wall is thicker, the dimensional limitation to CNTs is lower, and hence the calculated single layer thin wall conductivity is larger. It is noted from Figure 9d–f that the thin wall has much larger EM absorption than reflection. Although a single layer cell wall blocked a certain percentage of EM waves, a large number of cell walls in nanocomposite foam could fully absorb EM waves. Therefore, the conductive nanocomposite foam constructed by continuous cell walls has the characteristics of low EM reflection and high EM absorption.

**Figure 10:**
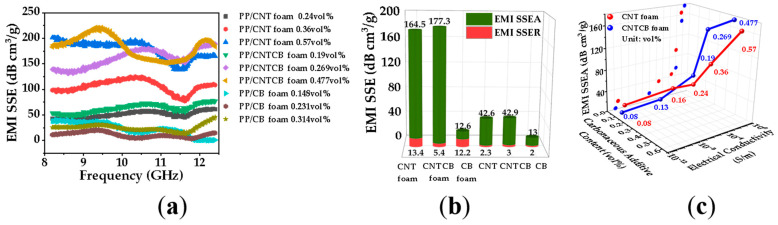
EMI SSE of PP/CNT, PP/CNTCB, and PP/CB conductive nanocomposite foam; (**a**) relationship between x-band frequency and EMI SSE at various carbon filler content (vol.%); (**b**) comparison between SSEA and SSER at initial nanocomposites’ carbon filler content; (**c**) relationship between carbon filler content, electrical conductivity and EMI SSEA.

Figure 10 shows that 0.48 vol.% PP/CNTCB conductive nanocomposite foam had the highest specific EMI shielding efficiency and specific EMI shielding absorption efficiency. As the rheological network in CNT system is easier to be formed at the same mass content of filler, the expansion ratio of PP/CNT nanocomposite foam is smaller than that of PP/CNTCB nanocomposite foam, so PP/CNTCB conductive nanocomposite foam has a higher specific shielding efficiency. Furthermore, from Figure 10a,b, PP/CB nanocomposites’ and foams’ specific EMI shielding efficiency was all much lower than other nanocomposites or foams. The results in Figure 10b show that the introduction of air and the 2D thin-wall structure significantly reduced the specific EM reflection efficiency, significantly enhanced the specific EM absorption efficiency, and markedly increased the specific EMI shielding efficiency. Figure 10c shows that with increasing the conductivity of nanocomposite foam, the specific EMI shielding efficiency increased.

**Figure 11:**
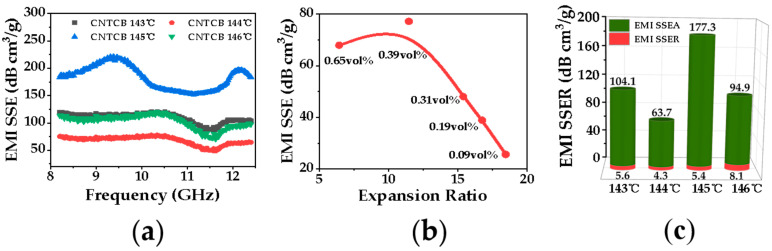
EMI SSE of PP/CNTCB conductive nanocomposite foams with different cellular structures; (**a**) relationship between x-band frequency and EMI SSE at various carbon filler content (vol.%); (**b**) relationship between expansion ratio and EMI SSE (at the same foaming condition of 144 °C and 15 MPa); (**c**) comparison between SSEA and SSER at initial nanocomposites’ carbon filler content.

Figure 11 presents the relationship between cellular structure and specific EMI shielding efficiency and observed the optimal specific EMI shielding efficiency. It was noted from Figure 11 that PP/CNTCB conductive nanocomposite foam has the best specific EMI shielding efficiency of 183 dB cm^3^/g when foamed at 145 °C and 15MPa. In conductive nanocomposite foam with low content of hybrid carbon fillers, due to the lesser degree of rheological network in the polymer matrix, cell growth is relatively easier. This can introduce a large amount of air. Therefore, nanocomposite foam’s density is markedly reduced which can promote the specific EMI shielding efficiency. However, the conductive network formed by carbon fillers is the key point for high EM absorption, so the optimum value of the nanocomposite foam’s specific EMI shielding efficiency exist, as shown in Figure 10b.

## 4. Conclusions

By using one-dimension 1D CNTs with a moderate aspect ratio to minimize the dimensional confinement from 2D thin walls and 0D CB with no dimensional confinement to connect the separated CNTs in thin walls and to expand the EM absorbing network, we constructed the main EM absorbing network with high EM absorption. By using scCO_2_ foaming, we obtained a cellular structure with multi-layer thin walls and a large amount of air cells to reduce the reflected EM. The nanocomposite foam with continuous 2D thin walls has low density, high conductivity and high EMI SSEA. When the volume content of hybrid conductive nanofillers is 0.48 vol.%, the obtained EMI shielding material had a specific EMI shielding performance as high as 183 dB cm^3^/g with 97% absorption and only 3% reflection and a density as low as 0.067 g/cm^3^.

## Data Availability

Not applicable.

## Supplementary Materials

The following are available online at https://www.mdpi.com/article/10.3390/polym13193278/s1, Figure S1: Numerical simulation of nucleation and growth of cells, Figure S2: Carbon nanofillers used in this work, Figure S3: Melting temperature curves of conductive nanocomposites, Figure S4: Crystallization temperature curves of nanocomposites, Figure S5: Primary heating curve of PP/CNTCB conductive nanocomposite foams with different cellular structures; (**a**) Melting curve under 143 °C, 15 MPa; (**b**) Melting curve under 145 °C, 15 MPa; (**c**) Melting curve under 147 °C, 15 MPa, Figure S6: SEM micrographs of CNTCB nanocomposite foams: (a1–a2) 143 °C, 5 wt% and 143 °C, 10 wt% CNTCB; (b1–b2) 144 °C, 5 wt% and 144 °C, 10 wt% CNTCB; (c1–c2) 145 °C, 5 wt% and 145 °C, 10 wt% CNTCB; (d1–d2) 146 °C, 5 wt% and 146 °C, 10 wt% CNTCB; (e1–e2) 147 °C, 5 wt% and 147 °C, 10 wt% CNTCB, Figure S7: Cellular structure of PP/CNTCB; (a) Cell size; (b) Expansion ratio; (c) Cell Density; (d) Cell wall thickness, Figure S8: High frequency dielectric property and EMI of single cell wall of PP/CNT, PP/CNTCB and CB conductive nanocomposite foams; (a) complex permittivity; (b) Dielectric loss; (c) EMI SE of single cell wall; (d) Asorptivity of single cell wall; (e) Reflectivity of Single cell wall; (f) Transmittance of single cell wall, Table S1: Foaming process conditions of PP/CNT, PP/CNTCB and PP/CB nanocomposites, Table S2: Different temperature conditions of PP/CNTCB foam, Table S3: Crystallization information of PP/CNT conductive nanocomposite, Table S4: Crystallization information of PP/CNTCB conductive nanocomposite, Table S5: Crystallization information of PP/CB conductive nanocomposite.
