# Supplementary material for: Synergistic Manipulation of Zero-Dimension and One-Dimension Hybrid Nanofillers in Multi-Layer Two-Dimension Thin Films to Construct Light Weight Electromagnetic Interference Material
Source: Polymers (Basel). 2021 Sep 26;13(19):3278. doi: 10.3390/polym13193278 (PMC8513059; doi:10.3390/polym13193278)
Supplement: Supplementary file 1 [file polymers-13-03278-s001.zip › polymers-1383608-supplementary.pdf]

Supporting Information

Synergistic Manipulation of Zero-Dimension and One-Dimension Hybrid Nanofillers in Multi-Layer Two-Dimension Thin Films to Construct Light Weight Electromagnetic Interference Material

1. Materials and Methods

1.1 Foaming process conditions

The foaming process conditions of nanocomposites were shown in **Table S1** and **Table S2** below.

Table S1. Foaming process conditions of PP/CNT, PP/CNTCB and PP/CB nanocomposites.

	Carbon filler Content (wt%)	Foaming temperature (℃)	Foaming pressure（MPa)
PP	0	145	15
CNT	3	145	15
	5	145	15
	8	145	15
	10	145	15
	15	145	15
CNTCB	3	145	15
	5	145	15
	8	145	15
	10	145	15
	15	145	15
CB	3	145	15
	5	145	15
	8	145	15
	10	145	15
	15	145	15

Table S2. Different temperature conditions of PP/CNTCB foam.

	Carbon filler Content (wt%)	Foaming temperature (℃)	Foaming pressure（MPa)
PP	0	143 144 145 146 147	15
CNTCB	3	143 144 145 146 147	15
	5	143 144 145 146 147	15
	8	143 144 145 146 147	15
	10	143 144 145 146 147	15
	15	143 144 145 146 147	15

1.2 Characterization

1.2.1 Transmission Electron Microscopy

Field emission Transmission Electron Microscopy (FE-TEM, Tecnai G2 F20 S-twin) was used to study the size and dispersive status of nano-scale carbonaceous additives, e.g. CB and CNT. Before observation, samples were sliced into 120 nm thick thin film in a cryogenic status.

1.2.2 Cellular Structure

The expansion ratio of conductive nanocomposite foams material could be measured by using density module and analytical balance.

Electron microscope images at different magnification were obtained by SEM test. The cell size information could be obtained by combining with *ImageJ* software. The cell density information could be obtained by formula (**S1**):

$$\mathrm{n}_{SEM} = \left(\frac{\mathrm{N}_{\mathrm{SEM\_}cross-\mathrm{sec}tion}}{A_{\mathrm{SEM\_}cross-\mathrm{sec}tion}} \right)^{\frac{3}{2}} \times EP, \qquad (\mathbf{S1})$$

where the n_{SEM} is the cell density (#/cm^3), $N_{SEM\text{-}cross\text{-}section}$ is the number of cellulars in the region of SEM diagram, $A_{SEM\text{-}cross\text{-}section}$ is the area of this region (cm^2), *N*/*A* is the cell density of this region (#/cm^2), and *EP* is the expansion ratio.

1.2.3 Electrical Conductivity

High Resistance Meter was used to test the resistance value of conductive nanocomposite and conductive nanocomposite foam, and the conductivity of the materials were calculated by formula (**S2**):

$$\sigma = \frac{\mathrm{L}}{\mathrm{R} \times \mathrm{S}}, \qquad (\mathbf{S2})$$

where the *L* is the length (m) along the conductive direction, *R* is the resistance (Ω) of the material, and *S* is the cross-sectional area (m^2) perpendicular to the conductive direction.

The volume content of CNT, CNTCB and CB (Vol_{CNT}, Vol_{CNTCB} *and* Vol_{CB}) could be obtained by the formulas (**S3**), (**S4**) and (**S5**) respectively:

$$\mathrm{Vol}_{CNT} = \frac{m \times wt_{CNT}}{\rho_{CNT} \times L \times W \times T}, \tag{S3}$$

$$\mathrm{Vol}_{CNTCB} = \frac{2m \times wt_{CNTCB}}{(\rho_{CNT} + \rho_{CB}) \times L \times W \times T}, \tag{S4}$$

$$\mathrm{Vol}_{CB} = \frac{m \times wt_{CB}}{\rho_{CB} \times L \times W \times T}, \tag{S3}$$

where M is the weight of the sample, wt_{CNT}, wt_{CNTCB} and wt_{CB} are the weigth percentage of CNT, CNTCB and CB, respectively. ρ_{CNT} and ρ_{CB} are the densities of CNT and CB respectively. L is the length of the sample, W is the width of the sample and T is the thickness of the sample.

1.2.4 Electromagnetic interference shielding

The detailed calculation procedures were as follows: reflection power coefficient (R), transmission power coefficient (T), absorption power coefficient (A), reflection SE (SE_R), absorption SE (SE_A) and total SE (SE_T) were calculated from scattering parameters.

The R, T and A could be calculated from scattering parameters (S_{11} and S_{21}) by following formulas:

$$R = |S_{11}|^2, \tag{S6}$$

$$T = |S_{21}|^2, \tag{S7}$$

$$A = 1 - R - T. \tag{S8}$$

SE_R, SE_A and SE_T could be obtained as follows:

$$SE_R = -10\lg(1-R), \tag{S9}$$

$$SE_A = -10\lg\left[T/(1-R)\right], \tag{S10}$$

$$SE_T = -10\lg T. \tag{S11}$$

1.3 Construction of conductivity model

In this model, the cellular structure was constructed by the Voronoi model, and the multidimensional solid packing model (Monte Carlo method) was used to construct the multidimensional packing conductive network. Combined with the conductive network model (Kirchhoff current law), the conductivity of conductive nanocomposite foam was quantitatively calculated.

Voronoi method was used to construct a three-dimensional (3D) cell model to simulate the nucleation and growth of the cell. The distribution of cellular nuclei in

volume element could be obtained by setting the number and spacing of cellular nuclei. 3D foaming materials with different cellular structures could be obtained by setting parameters such as different expansion rate and cell size in the model, as shown in **Figure S1**.

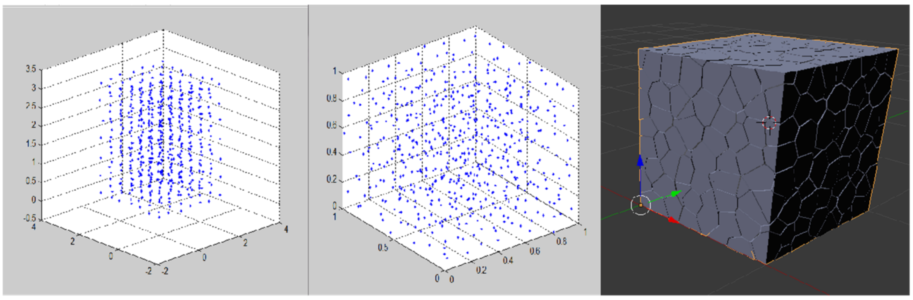

(a) (b) (c)

Figure S1. Numerical simulation of nucleation and growth of cells.

After constructing 3D cell, due to the isolation effect of cell, multidimensional mixed conductive fillers are continuously distributed on two-dimensional (2D) cell wall. In the program, Monte Carlo method is used to selectively distribute multidimensional mixed conductive fillers on the cell walls. For foaming materials, 2D cell wall is 2D stretched during the process of the growth of the cell core into a cell, so that the Angle between one-dimensional (1D) CNTs and one side changes, resulting in 2D orientation of 1D CNTs. Therefore, 1D CNTs are selectively distributed in the cell wall.

The introduction idea of 1D CNTs was as follows:

The position of the center point (x, y, z) could be expressed as follow:

$$\mathrm{x} = L_x \times rand \text{ ,} \tag{S12}$$

$$y = L_y \times rand \text{ ,} \tag{S13}$$

$$z = L_z \times rand \text{ .} \tag{S14}$$

Polar Angle (θ_i) and azimuth (φ) could be expressed as follow:

$$\theta_i = \pi \times rand \text{ ,} \tag{S15}$$

$$\varphi_i = \arcsin(\delta \div \frac{\pi}{2}) \text{ .} \tag{S16}$$

The introduction idea of 0D CBs was as follows:

The position of the center point (x, y, z) could be expressed as follow:

$$x = L_x \times rand\,, \quad \textbf{(S17)}$$

$$y = L_y \times rand\,, \quad \textbf{(S18)}$$

$$z = L_z \times rand\,. \quad \textbf{(S19)}$$

The conductive network consists of junction resistance R_j (the tunneling resistance of two conductive nanofibers within a certain distance) and segment resistance R_C (the intrinsic resistance of the conductive part of the conductive nanofiber)[1]. The R_c were calculated by formula (**S20**):

$$R_c = \frac{4L_c}{\sigma_{CNT}\pi D^2}\,, \quad \textbf{(S20)}$$

where L_c is the length of the CNT segment involved in conducting, σ_{CNT} is the intrinsic conductivity of the conducting nanofiber, and D is the diameter of the conducting nanofiber. The R_j were calculated by formula (**S21**):

$$R_j = \frac{v}{A \times J} = \frac{h^2 \times d}{A \times e^2 \times \sqrt{2\mathrm{m}\lambda}} e^{\left(\frac{4\pi \times d}{h} \times \sqrt{2\mathrm{m}\lambda}\right)}\,, \quad \textbf{(S21)}$$

where d is the shortest distance between CNTs, v is the electrical potential difference, j is the tunnelling current density, A is the cross-sectional area of the tunnel (approximated as D^2), e is the elementary charge, m is the mass of an electron, h is Planck's constant, and λ is the height of the barrier (taken as the work function of the CNT, 5.0 eV).

The current matrix is calculated by Kirchhoff's current law, and the equation is as follows:

$$\begin{bmatrix} -\sum g_{1,..} & g_{12} & \cdots & g_{1n} \\ g_{21} & -\sum g_{2...} & \cdots & g_{2n} \\ \vdots & \vdots & \ddots & \vdots \\ g_{n1} & \cdots & \cdots & -\sum g_{n,..} \end{bmatrix} \cdot \begin{bmatrix} u_1 \\ u_2 \\ \cdots \\ u_{n-1} \end{bmatrix} = \begin{bmatrix} 1 \\ 0 \\ \cdots \\ -1 \end{bmatrix}. \quad \textbf{(S22)}$$

After the voltage matrix is obtained, according to Ohm's law, the resistance of the network is equal to the voltage difference between the two ends of the network divided by the current value flowing through the network (current value is 1). That is:

$$R_N = \frac{|U_1 - U_{n-1}|}{1}\,. \quad \textbf{(S23)}$$

The network conductivity thus obtained is:

$$\rho = \frac{R_N \times S}{L}\,. \quad \textbf{(S24)}$$

2. Results

2.1 CNT and CB Nanofillers

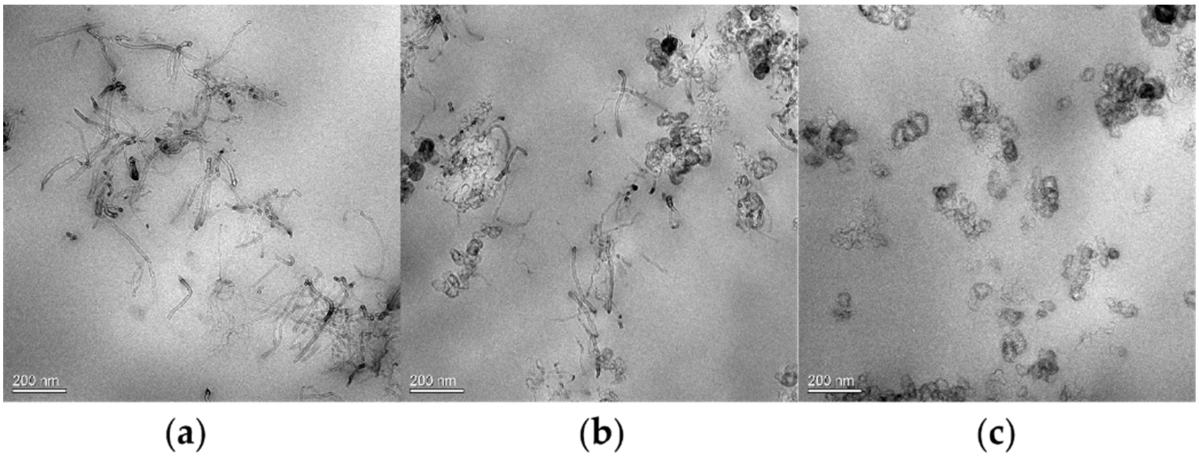

Figure S2. Carbon nanofillers used in this work.

2.2 Thermal property of conductive nanocomposites

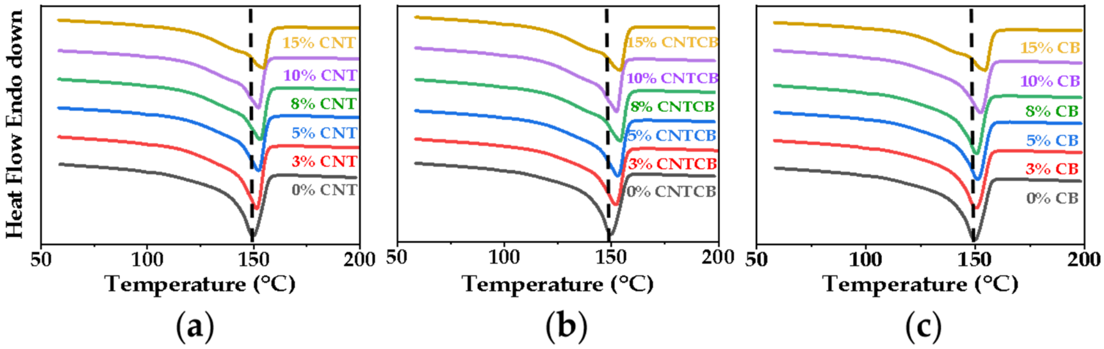

Figure S3. Melting temperature curves of conductive nanocomposites.

The melting temperatures of PP/CNT, PP/CNTCB and PP/CB conductive nanocomposites were shown in **Figure S3**. The melting temperature of the three kinds of conductive nanocomposites with different packing ratio moved to high temperature with the increase of packing content.

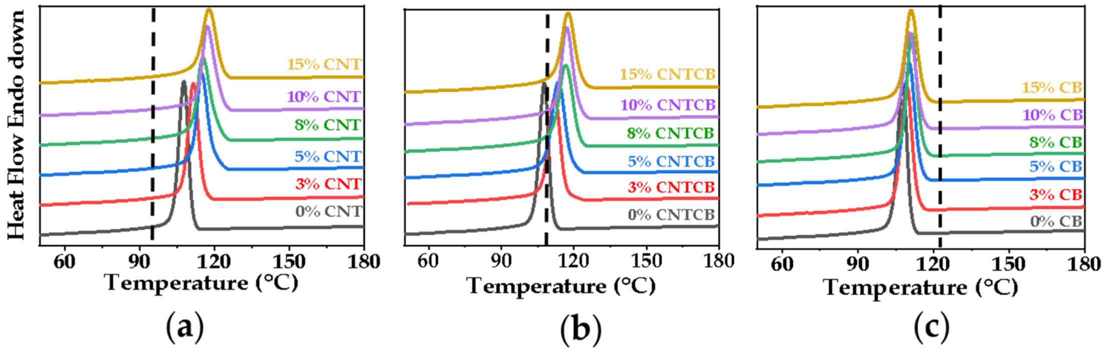

Figure S4. Crystallization temperature curves of nanocomposites.

The crystallization temperature of PP/CNT, PP/CNTCB and PP/CB conductive nanocomposites were shown in **Figure S4**. The crystallization temperature of the three kinds of conductive nanocomposites with different packing ratio moved to high temperature with the increase of packing content.

Table S3. Crystallization information of PP/CNT conductive nanocomposite.

CNT Content (wt%)	T_c (℃)	T_m (℃)	Crystallinity(%)
3	112.0	151.4	28.0
5	114.4	153.0	30.5
8	116.0	153.3	30.5
10	116.7	153.5	29.9
15	116.8	154.6	29.6

Table S4. Crystallization information of PP/CNTCB conductive nanocomposite.

CNTCB Content (wt%)	Tc (℃)	Tm (℃)	Crystallinity(%)
3	115.1	151.8	27.7
5	115.1	152.7	25.4
8	115.6	153.3	27.0
10	117.4	153.5	26.9
15	118.0	154.7	25.7

Table S5. Crystallization information of PP/CB conductive nanocomposite.

CB Content (wt%)	Tc (℃)	Tm (℃)	Crystallinity(%)
3	109.4	150.3	25.5
5	110.7	151.5	26.4
8	111.4	150.9	27.2
10	111.6	151.1	27.0
15	111.3	153.0	26.6

Table S3, **Table S4** and **Table S5** summarized the crystallization temperature, melting temperature and crystallinity data of PP/CNT PP/CNTCB and PP/CB conductive nanocomposites. The results showed that the crystallinity of the three kinds of conductive nanocomposites increased first and then decreases with the increase of the content of conductive filler. May cause is the CNT and CB nanofillers have played an important role of heterogeneous nucleation, this promotes the crystallinity increase, while the addition of CNT and CB nanofillers constructed rheological network will improve the molecular chain rigidity, this makes it harder for chains to fit into the lattice, the more significant of the trend, which has suppressed the promotion of crystallinity.

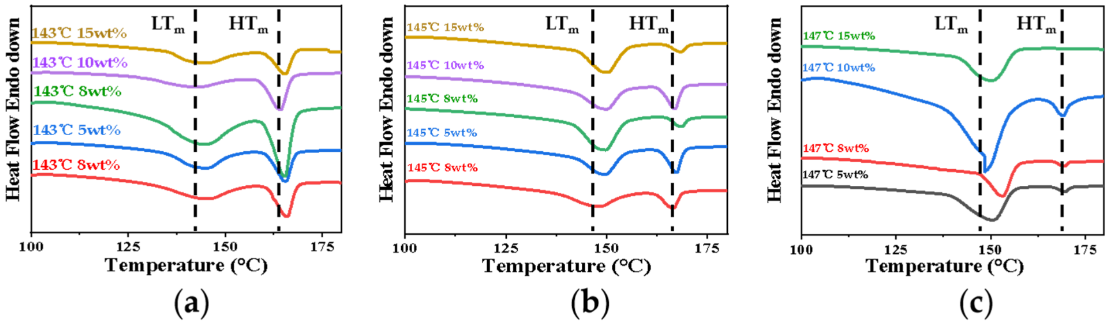

Figure S5. Primary heating curve of PP/CNTCB conductive nanocomposite foams with different cellular structures; (**a**) Melting curve under 143℃, 15 MPa; (**b**) Melting curve under 145℃, 15 MPa; (**c**) Melting curve under 147℃, 15 MPa.

2.3 SEM image of PP/CNTCB conductive nanocomposite foams under the different process conditions

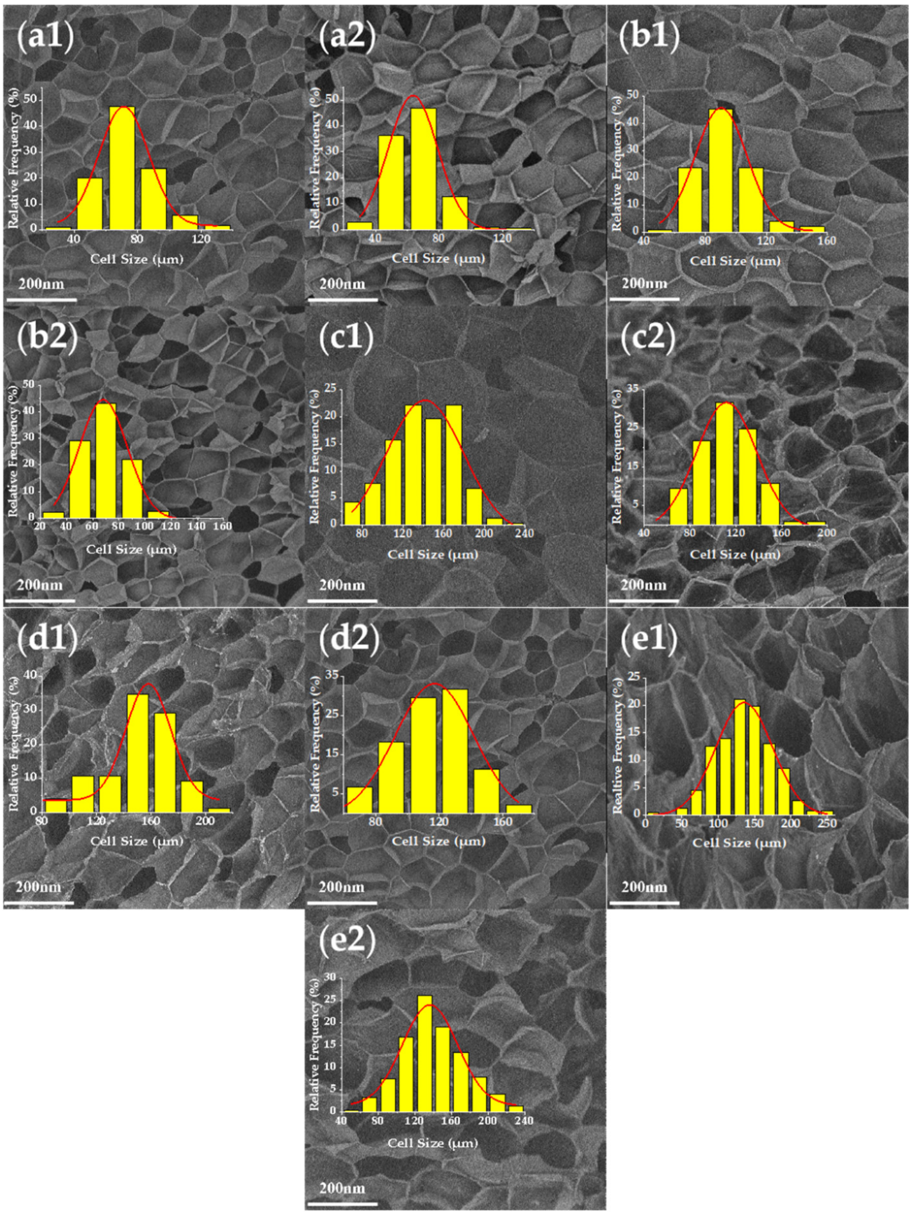

Figure S6. SEM micrographs of CNTCB nanocomposite foams: (**a1** – **a2**) 143℃, 5 wt% and 143℃, 10 wt% CNTCB; (**b1** – **b2**) 144℃, 5 wt% and 144℃, 10 wt% CNTCB; (**c1** – **c2**) 145℃, 5 wt% and 145℃, 10 wt% CNTCB; (**d1** – **d2**) 146℃, 5 wt% and 146℃, 10 wt% CNTCB; (**e1** – **e2**) 147℃, 5 wt% and 147℃, 10 wt% CNTCB.

2.4 Cellular structure of nanocomposite foams

To obtain different cellular structures of PP/CNTCB and explore the influence of cellular structure on material conductivity, conductive nanocomposite foam of PP/CNTCB with different cellular structures were prepared by adjusting different molding processes, as shown in **Figure S7** (**a**) - (**d**). At 143℃ and 144℃, a large number of heterogeneous interfaces between the amorphous and crystalline regions can be used for nucleation, so the content of carbon filler has little effect on the cell density. At 145℃ and 146℃, the heterogeneous interface between the crystalline

region and the amorphous region is less, and the carbon filler content has a great influence on the cell density (the higher the filler content is, the higher the cell density is). When the crystal region is nearly completely fused to the point that the cell structure cannot be maintained (147℃), although the higher the filler content is, the higher the cell density is, the higher the filler content is, the greater the elasticity of the matrix is, and the collapse of the cells leads to the consolidation of the cells (the observed decrease in the number of cells, the corresponding decrease in the calculated cell density). This is also why the expansion ratio at 147℃ increases with the filler first (strength increases to a certain extent) and then decreases (cell collapse).

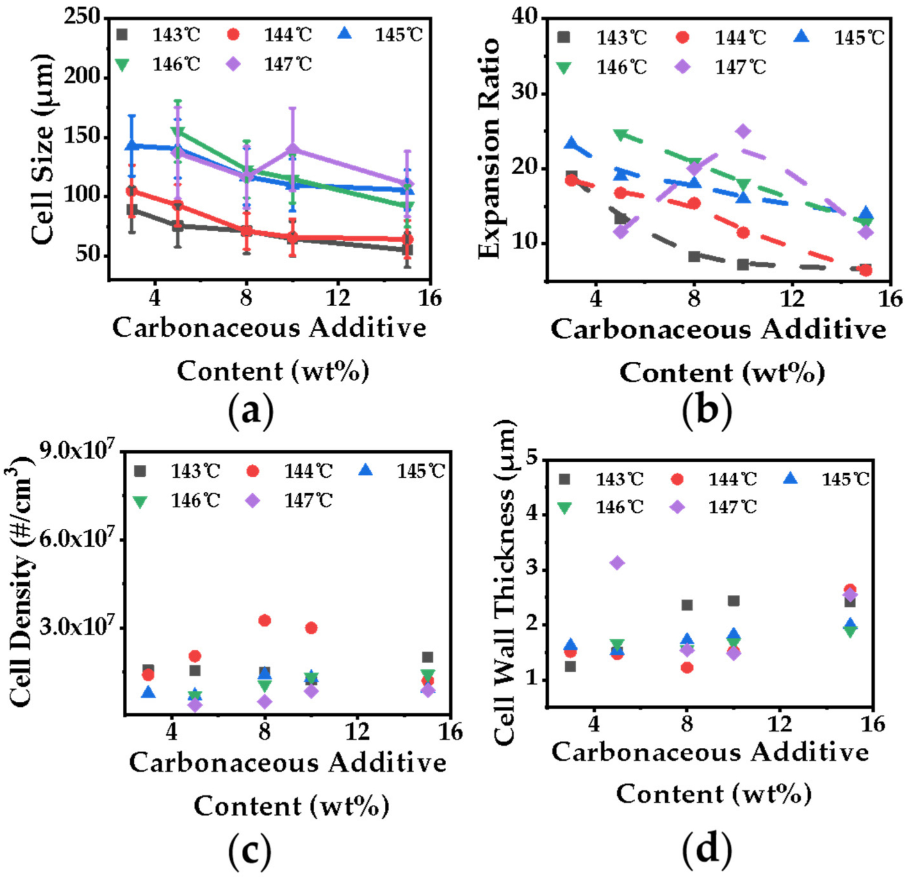

Figure S7. Cellular structure of PP/CNTCB; (**a**) Cell size; (**b**) Expansion ratio; (**c**) Cell Density; (**d**) Cell wall thickness.

2.5 High frequency dielectric properties of conductive nanocomposite foam

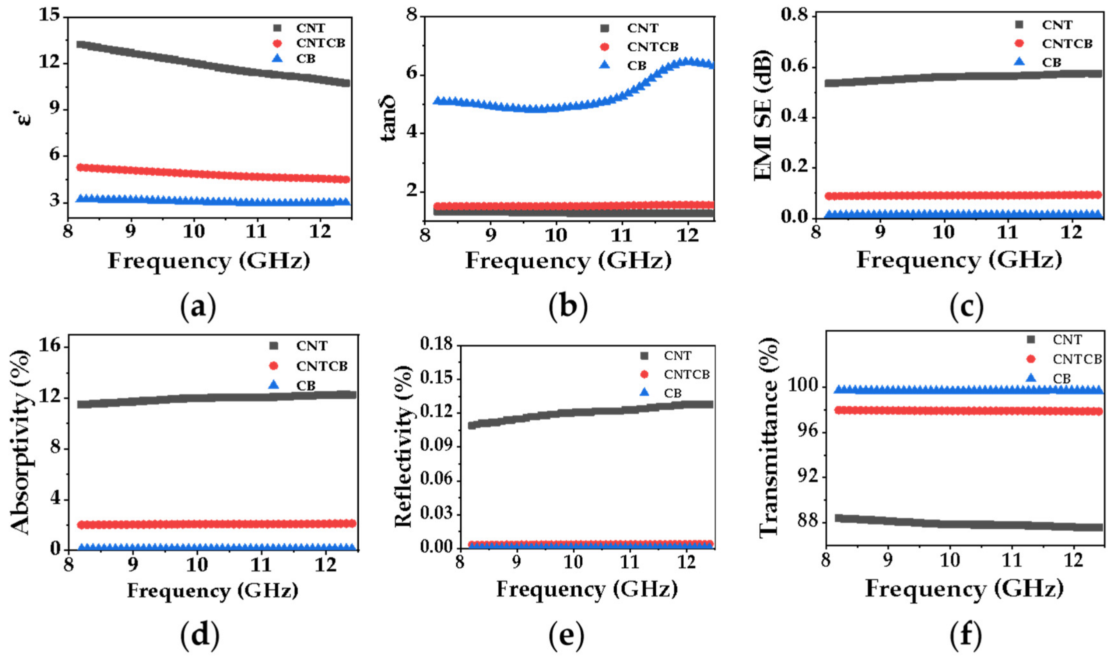

Figure S8. High frequency dielectric property and EMI of single cell wall of PP/CNT, PP/CNTCB and CB conductive nanocomposite foams; (**a**) complex permittivity; (**b**) Dielectric loss; (**c**) EMI SE of single cell wall; (**d**) Asorptivity of single cell wall; (**e**) Reflectivity of Single cell wall; (**f**) Transmittance of single cell wall.

Reference

1. Wang, Z.F.; Ye, X. A numerical investigation on piezoresistive behaviour of carbon nanotube/polymer composites: mechanism and optimizing principle. *Nano.* 2013, 24 (26), 265704.
